# Supplementary material for: Change in population structure, policy adjustment, and China’s public pension sustainability
Source: PLoS One. 2025 Sep 26;20(9):e0331739. doi: 10.1371/journal.pone.0331739 (PMC12469360; doi:10.1371/journal.pone.0331739)
Supplement: Attachment — Submitted filename: Review_PONE-D-24-42892.pdf [file pone.0331739.s002.pdf]

Review PONE-D-24-42892

This paper deals with predictions for the public pension fund of urban employees in China under various assumptions about fertility, economic growth and policy decisions on retirement age and pension growth rate. The results look interesting.

However, the analysis/elaboration suffers from important shortcomings, such as details on the actuarial model used and the methodology applied to derive the projections. Also missing is an explanation of the public pension system in China and a justification of why only urban workers are analysed.

Specific remarks:

1) The paper concentrates specifically in China. This should be incorporated in the tittle.
2) The paper states that the analysis is based on actuarial models for pension income, expenditure and accumulated balance, but does not make explicit or explain what these actuarial models are; in particular, what actuarial tables on future survival/mortality are used?
3) Does immigration/emigration play no role in the evolution of the population in China? This aspect needs to be explained.
4) The literature review in the Introduction section is too generic and should be more specific. On the other hand, the terminology used, 'pension funds', has different interpretations depending on the pension system in each country. The characteristics of the public pension system in China should be explained succinctly, which, from the subsequent development, seems to use only the pay-as-you-go system, so the term 'pension fund' may not be the most appropriate.
5) In Section 2. First paragraph. The definition of the four groups into which the participants are divided is not clear and needs to be better explained, it is not sufficient to cite Documents No 26, 1997 and 38, 2005. Issues such as, for example, should be made clear:
   - A person from the post-1997 employees group can also be a member of the post-2005 retiree group?
6) Can a person from the post-1997 retirees also be a member of the post-2005 retiree group?
7) In formula (1):
   - Where does t start?
   - What is t_0?
   - If AI_t represents the pension income, for t>1997, why do we include the group i=1 which collects pre-1997 retirees?
8) In formula (2):
   - Where does the variable t begin and end?
   - $\bar{B}^i_{t,x}$ and $s^i_{t,x}$ depend on x?
   - The product $\prod$ should end at $t-1$.
9) In formula (3):
   - Where does the variable t begin and end?
   - $\bar{B}^i_{t,x}$ and $s^i_{t,x}$ depend on x?
   - Is it possible that $1998-(t-x+a_t)$ becomes negative?
   - The product $\prod$ should end at $t-1$.
10) In formula (4), r should be defined.

11) Subsection 2.4:
    - The assumption that “the age at first employment of urban employees is 20 years” should be better justified, perhaps using official statistics. This age is constant for all of the years included in the study?
    - Which is the difference between “female workers” and “female cadres”?
    - Given the large difference in the retirement ages of men and women, do expressions (1) and (2) take into account that the parameters a_t and b_t are different for each sex? How is this issue implemented in the calculations? Are the same parameters used for both sexes?
    - The assumption that “the age at the average retirement age of urban employees is 55 years” should be better justified, perhaps using official statistics.
    - The cohort component method used to predict the population distribution by age in urban and rural areas must be explained, at least in outline, and the predictions obtained should be shown in an annex.
    - The number of pension contributors, the age distribution of pension recipients and the labor participation rates of corresponding age groups and also the number of pension contributors and pension recipients in the future. All these results should be shown in an annex.
    - Why only two future fertility scenarios, and why not include a scenario of declining fertility?
    - What are the values of c_t?
12) Section 3. What is the ‘change in the population structure’ ? Describe this change.
13) The authors should consider changing the presentation of the results, replacing some tables with graphs and leaving the tables in an annex. It would also be useful to create more elaborate tables that include summarised information: for example, first year of deficit occurrence according to the scenario.
14) The introduction mentions different articles that make predictions about the China's pension fund gap. The results obtained in this paper should be compared with those of previous work.
15) Although I have not checked the rest of the references, references 18, 23, 32 and 34 are incorrect.
