# Supplementary material for: Change in population structure, policy adjustment, and China’s public pension sustainability
Source: PLoS One. 2025 Sep 26;20(9):e0331739. doi: 10.1371/journal.pone.0331739 (PMC12469360; doi:10.1371/journal.pone.0331739)
Supplement: Attachment — Submitted filename: 0727-Response to reviewers.docx [file pone.0331739.s003.docx]

**Response to reviewers**

**Dear Editor,**

We sincerely thank you and the reviewers for the thorough evaluation of our manuscript and the constructive suggestions provided. We have carefully revised the manuscript in response to all comments, improving its clarity, rigor, and completeness. Below we summarize the major revisions made in response to each reviewer’s feedback.

**Response Summary**

**Revisions Based on Reviewer 1’s Comments**

1. **Clarification of “high fertility rate”**
   We added a clear definition at the first mention: the “high fertility rate” scenario refers to the fertility level under China’s universal two-child policy. Following Lu & Cai (2016), a theoretical TFR of 1.94 is adopted as a hypothetical scenario for simulation purposes only, and we explicitly note that actual TFR levels remain much lower.

**Revisions Based on Reviewer 2’s Comments**

1. **Title modification**
   Revised to: “Change in Population Structure, Policy Adjustment, and China Public Pension Sustainability.”
2. **Detailed actuarial model explanation**
   A full description of the three core modules (Pension Revenue, Pension Expenditure, and Cumulative Balance Models) has been added, with explicit variable definitions and references to China’s National Life Tables (2010 & 2020).
3. **Migration explanation**
   We clarified that migration was excluded due to the national-level policy focus but acknowledged its importance for future research.
4. **First employment age assumption**
   Provided justification based on legal, educational, and modeling considerations, confirming that a fixed age of 20 years balances realism and model simplicity.
5. **Definition of female workers vs. female cadres**
   Clarified based on occupational distinctions under China’s retirement system.
6. **Average retirement age assumption**
   Justified using official MOHRSS data (average 54+, rounded to 55 years for modeling).
7. **Population projections**
   Added a brief description of the cohort-component method.
8. **Fertility scenarios**
   Clarified that only baseline (TFR=1.6) and high fertility (TFR=1.94) scenarios were set due to policy relevance.
9. **Presentation improvements**
   Converted key tables to graphs, and added comparisons with previous studies in Section 4.3.
10. **References**
    Corrected formatting errors, especially references 18, 23, 32, and 34.

**Revisions Based on Reviewer 3’s Comments**

1. **Clarification of the “10% GDP subsidy” statement**
   Explained its legal basis (Social Insurance Law, Article 13), fiscal responsibility (MOHRSS statements), and clarified it as a worst-case warning from James (2002) rather than a deterministic forecast.
2. **Fertility policy evaluation**
   Added detailed analysis showing that increased fertility has short-term fiscal costs (extra CNY 769.3 bn/year deficit pre-2041) and only modest long-term benefits (12% deficit reduction by 2050), emphasizing the need for parametric reforms.
3. **Four-group classification**
   Explained its policy basis (State Council Documents No.26 (1997) and No.38 (2005)) and its purpose to quantify transition costs.
4. **Formula 1 clarification**
   Defined all variables and justified summations as necessary for group and age aggregation, with heterogeneity reflected indirectly.
5. **Macro vs. micro data**
   Justified the use of macro-aggregated data due to data access limits and the study’s focus on long-term sustainability.
6. **Contribution base growth rate**
   Clarified it as an assumed parameter, supported by policy documents and academic research linking wage and GDP growth.
7. **Cumulative balance model**
   Explained that no discounting was applied as the focus is on cash flow solvency, with present-value analysis reserved for future work.
8. **Policy implications**
   Highlighted gradual delayed retirement as the most feasible reform, consistent with China’s 2025 Progressive Retirement Law (surplus of CNY 49.44 tn by 2050 under gradual delay).

**Conclusion**

We have revised the manuscript substantially to address all reviewers’ comments. The revised version now provides clearer model definitions, improved methodological transparency, and enhanced policy relevance. We thank you and the reviewers again for your valuable feedback, which has greatly improved the quality of our work.

**Best regards,**
[Meng Jia]

**Response to Reviewer 1**

Dear Reviewer,

Thank you very much for your thoughtful and encouraging comments on our manuscript. We truly appreciate your recognition of our work and your constructive suggestion, which will help us improve the clarity of the paper.

Regarding your concern about the definition of the “high fertility rate” scenario:

We agree that the definition should be clarified at the beginning of the manuscript, as a total fertility rate (TFR) of 1.94 may not generally be considered “high” in many parts of the world, but it has specific contextual meaning in China. In the revised manuscript, we have added a clear definition when the term is first introduced. Specifically, we state that in this study, the “high fertility rate” scenario refers to the fertility level under the universal two-child policy in China, under which families are allowed to have up to two children. Based on the simulation assumptions of Lu and Cai (2016) (From Demographic Dividend to Reform Dividend: A Simulation Based on China’s Potential Growth Rate, Population & Economics, 39, 3–23), we adopt a theoretical TFR of 1.94 to represent this “high fertility” scenario. We also explicitly note that this is a hypothetical assumption for theoretical discussion only, and that the actual TFR in China is expected to remain far below this level.

We have revised the corresponding section accordingly to ensure that readers can easily understand the context-specific meaning of “high fertility rate.”

Thank you again for your valuable comment.

**Response to Reviewer 2**

Dear Reviewer,

Thank you very much for your careful reading and valuable comments on our manuscript. Your suggestions are constructive and have greatly helped us improve the clarity, rigor, and completeness of the paper. We have carefully addressed each point, and the manuscript has been revised accordingly. Our detailed responses are as follows:

**1. Title should explicitly indicate that the study focuses on China**

**Response:** The title has been revised as suggested. The new title is:

*“Change in Population Structure, Policy Adjustment, and China Public Pension Sustainability.”*

**2. Clarification of the actuarial model and mortality/life tables used**

**Response:** A detailed explanation of the actuarial model has been added in Section 2, specifying its three core modules:

1. Pension Revenue Model

The annual pension revenue is calculated based on the number of contributors, wage growth rate, and contribution rate. The formula incorporates age-specific survival rates to estimate the number of active insured individuals $N_{t,x}^{i}$.

${AI}_{t}=[\sum_{i=1}^{4} \sum_{x=a_{t}}^{b_{t}-1} N_{t,x}^{i}]\times\bar{w}_{t}\times R_{t}$（1）

where $N_{t,x}^{i}$ is estimated based on age-specific surviving population projections.

2. Pension Expenditure Model

The calculation of pension expenditure involves the survival years of retirees, pension growth rate, and benefit level, which require age-specific mortality rates to compute the survival rate of retirees.

${AC}_{t,b}=\sum_{i=1}^{4} \sum_{x=b_{t}}^{c_{t}} [N_{t,x}^{i}\times\bar{B}_{t,x}^{i}{\times s}_{t,x}^{i}\times\prod_{s=t-x+b_{t}}^{t} (1+g_{s})]$(2)

${AC}_{t,g}=\sum_{i=2}^{3} \sum_{x=b_{t}}^{c_{t}} {\{N}_{t,x}^{i}\times\bar{G}_{t,x}^{i}{\times\left[ 1998-\left( t-x+a_{t} \right) \right]\times v}_{t,x}^{i}\times\prod_{s=t-x+b_{t}}^{t} \left( 1+g_{s} \right)\}$(3)

Here, the age-specific number of retirees $N_{t,x}^{i}$ is directly derived from life tables.

3. Cumulative Balance Model

The cumulative pension balance is dynamically updated by iterating the revenue-expenditure difference, relying on the outputs of the previous two models. The assumptions of survival rates and mortality rates remain crucial in this module.

$F_{t}=F_{t-1}\left( 1+r \right)+\left( {AI}_{t}-{AC}_{t} \right).$ (4)

where $\mathrm{AI}_{t} \mathrm{and}\mathrm{AC}_{t}$ are calculated based on the active and retired population sizes adjusted by mortality rates.

We specify that age-specific mortality rates are based on China’s National Population Census Life Tables (2010 and 2020 editions), with interpolations to estimate annual survival probabilities. Variables and formulas (1)–(4) are now fully defined in the text.

**3. Explanation for excluding migration in the model**

**Response:** Migration was not incorporated into the core analytical model of this study; however, this does not imply that migration plays “no role” in China’s demographic evolution. The exclusion of migration primarily reflects the scope and focus of the present research.

The central objective of this paper is to evaluate the impact of population structure changes (aging and fertility rates) and domestic policy adjustments (delayed retirement and pension growth rates) on the sustainability of China’s Urban Employee Basic Pension (UEBP) system. Accordingly, all model parameters are set at the national level, based on macro variables such as fertility rates, statutory retirement ages, and economic growth rates. Regional variations in migration patterns and the influence of population mobility on the composition of insured participants were not considered. Specifically, the number of contributors in the actuarial model（$N_{t,x}^{i}$) is projected solely based on age distribution and fertility assumptions, without incorporating cross-regional migration variables. The macro-policy scenarios designed in this study are limited to fertility policy, retirement age, pension growth rate, and economic growth, and no migration scenarios were simulated.

That said, numerous empirical studies have demonstrated that population mobility significantly affects China’s demographic dynamics and the regional sustainability of pension systems by reshaping population structures, labor supply, and the distribution of aging. Recognizing this limitation, future research will seek to integrate migration factors to enhance the accuracy and policy relevance of pension sustainability simulations.

**4. Assumption of first employment age at 20**

**Response:** The assumption that the first employment age of urban employees is 20 years is based on a combination of legal requirements, educational realities, and the need for model simplification. This age remains fixed throughout the study period and is not dynamically adjusted across historical stages, for the following reasons:

Legal and educational constraints

The statutory minimum employment age in China is 16 years (Labor Law, Article 15). However, the actual labor market entry age has increased significantly due to the universalization of compulsory education and the expansion of higher education.

Extended education duration: Most individuals enter the labor market after completing high school or vocational education, typically around 18 years of age.

Job-seeking transition period: Adding a transitional period for job seeking, assuming a stable first employment age of 20 years is consistent with the current labor market reality.

Model simplification and consistency

Rationale for a fixed parameter: Actuarial forecasting requires controlling for certain parameters to isolate policy effects (e.g., delayed retirement, fertility changes). Allowing the first employment age to vary over time would unnecessarily increase model complexity.

Limited historical variation: Although the average working-age population increased from 32.25 years in 1985 to 39.72 years in 2022, changes in the first employment age have been relatively small (e.g., only a 1–2 year delay for post-2000 cohorts compared to post-1990 cohorts), exerting negligible impact on long-term pension sustainability projections.

In summary, assuming a fixed first employment age of 20 years strikes a balance between the legal minimum age (16 years), educational attainment (post-high school entry), and the need for model simplicity. While this assumption does not fully account for urban–rural or gender differences or minor historical fluctuations, it is unlikely to affect the robustness of the long-term pension sustainability results and remains broadly consistent with the dominant trend in China’s labor market.

**5. Difference between “female workers” and “female cadres”**

**Response:** According to China’s current retirement system, the distinction between female workers and female cadres primarily lies in the nature of their positions:

Female workers (non-managerial positions): This category refers to women engaged in production, operational, or service-oriented roles that are non-managerial in nature, such as assembly line workers, cashiers, or logistics and support staff.

Female cadres (managerial/technical positions): This category refers to women holding managerial or professional technical positions with decision-making authority or specialized technical functions, such as department managers, financial supervisors, engineers, or teachers.

**6. Average retirement age assumption (55 years)**

Response: The assumption is based on the statement by Vice Minister Zhang Yizhen (Ministry of Human Resources and Social Security), who reported that the average retirement age of urban employees is slightly above 54 years. For modeling simplicity, we rounded it to 55 years.

**7. Population projection method & results**

**Response:** We added a brief explanation of the cohort-component method used for projecting age-specific population distributions in Section 2.4.

**8. Fertility scenarios**

**Response:** We clarified that only baseline (TFR = 1.6) and high fertility (TFR = 1.94) scenarios were set to evaluate policy interventions. A lower fertility scenario was not included because it had limited policy relevance at the time of the study.

**9. Presentation of results & comparison with previous studies**

**Response:** We converted part of the tables into graphs for better readability, and added a comparison with previous studies in Section 4.3.

**10. Reference corrections**

**Response:** We thoroughly checked and corrected all reference formatting errors, including references 18, 23, 32, and 34.

**Response to Reviewer 3**

Dear Reviewer,

Thank you very much for your detailed and insightful comments. We have carefully revised the manuscript and provide the following responses to your specific questions, maintaining as much detail as possible for clarity.

**1. Clarification of the statement “China will need to subsidize pension deficits equivalent to 10% of GDP”**

The statement originates from James (2002) and should be interpreted in the context of legal obligations, government roles, and the nature of the projection:

**(1) Legal basis and fiscal responsibility**

The Social Insurance Law of China (Article 13) clearly stipulates that “when the basic pension fund is insufficient to cover payments, the government shall provide subsidies.” This legal provision makes fiscal subsidies a mandatory state responsibility, not a voluntary or merely contractual arrangement.

Furthermore, the Ministry of Human Resources and Social Security (MOHRSS) has officially stated that historical pension liabilities are jointly borne by the social insurance system and the national budget, with fiscal transfers used to fill the funding gap. Therefore, government subsidies are a statutory fiscal safety net rather than an ad hoc intervention.

**(2) Social contract dimension**

China’s public pension system embodies an intergenerational social contract: the working-age population finances current retirees, and the government, as the institutional guarantor, ensures system sustainability.

When demographic aging undermines the balance of the pay-as-you-go (PAYG) system (e.g., our baseline projection indicates a cumulative deficit of CNY 147.4 trillion by 2050), fiscal subsidies become necessary to maintain this social contract. However, the contract is institutionalized through law, making it a state obligation rather than simply a moral or political commitment.

**(3) Nature of the “10% of GDP” projection**

James’s estimate assumes a no-reform scenario (low statutory retirement age, high replacement rates) and represents a worst-case risk warning rather than a deterministic outcome.

Our simulations show that policy adjustments—such as raising the statutory retirement age to 65 or controlling pension growth rates to ≤0—can effectively avoid such extreme deficits. Thus, the 10% of GDP figure should be seen as a cautionary assumption rather than a predicted reality.

**2. Is increasing fertility always beneficial to pension sustainability?**

While raising fertility can improve pension sustainability in the long run, it is not always beneficial and carries significant short-term fiscal costs and structural contradictions:

**(1) Limitations: short-term deterioration and modest long-term effects**

Short-term fiscal deterioration: Newborns remain purely dependent consumers for roughly 20 years, requiring childcare subsidies, education, and healthcare, while not contributing to the labor force or pension system.

Our simulations show that under a high-fertility scenario (TFR = 1.94), the cumulative pension deficit is actually larger than in the baseline (TFR = 1.6) before 2041, with an average annual deficit increase of CNY 769.3 billion.

Limited long-term improvement: Even with TFR raised to 1.94, the cumulative deficit in 2050 is reduced by only 12%, and the first deficit year (2028) remains unchanged. The positive labor supply effects only emerge after 2041 and are insufficient to offset accumulated debts by 2050.

**(2) Policy paradox: “one step forward, one step back”**

Historical structural contradictions: The one-child policy in the 1980s temporarily reduced population pressure but caused a sharp decline in the working-age population and a surge in the old-age dependency ratio (2.3:1 in 2020, projected 0.8:1 in 2050). Encouraging births now is essentially a corrective measure, but new labor entrants will take decades to fill the gap, creating policy-cycle fluctuations.

Competition for fiscal resources: Pronatalist policies (e.g., Hohhot’s CNY 100,000 subsidy for third-child families) directly compete with pension subsidies, potentially reducing the fiscal space available for pension deficit coverage in the short term.

Thus, increasing fertility is a necessary but insufficient response to aging. Without parametric reforms (e.g., delayed retirement, controlled benefit growth), fertility adjustments risk creating a cycle of higher childcare costs aggravating current deficits, with future gains insufficient to offset accumulated debt.

**3. Basis for the four-group classification of pension participants**

The classification of participants into (1) pre-1997 retirees, (2) post-1997 retirees, (3) post-2005 retirees, and (4) post-1997 employees is based on China’s pension reform milestones, particularly:

State Council Document No.26 (1997): Transition from enterprise-based PAYG to a mixed system combining social pooling + individual accounts.

State Council Document No.38 (2005): Refined benefit calculation and introduced transitional pensions to compensate for pre-reform service years.

The classification captures systemic differences in benefit rules rather than international “best practice.”

Pre-1997 retirees: Fully under the old PAYG system, no individual accounts.

Post-1997 retirees: Subject to transitional policies, combining basic and transitional pensions.

Post-2005 retirees: Fully covered by the new formula, directly linked to contribution years and individual accounts.

Post-1997 employees: The main contributors under the new system.

This classification allows for precise quantification of transition costs and separation of historical debt vs. new-system liabilities. Although international experience (e.g., Germany’s transitional compensation, UK’s three-tier system) may have inspired design ideas, the grouping is fundamentally a product of China’s policy evolution.

**4. Pension revenue model (Formula 1): variable definitions and the use of summations**

The pension revenue model is defined as:

$${AI}_{t}=[\sum_{i=1}^{4} \sum_{x=a_{t}}^{b_{t}-1} N_{t,x}^{i}]\times\bar{w}_{t}\times R_{t}=[\sum_{i=1}^{4} \sum_{x=a_{t}}^{b_{t}-1} N_{t,x}^{i}]\times\bar{w}_{t_{0}-1}\times\prod_{s=t_{0}}^{t} \left( 1+k_{s} \right)\times R_{t}$$

Variable definitions:

${AI}_{t}$: pension revenue in year 𝑡;

i: group classification (1–4 as defined above);

$a_{t}$and $b_{t}$: ages of labor market entry and retirement in year 𝑡;

$\sum_{i=1}^{4} N_{t,x}^{i}$: number of contributors of age 𝑥 in group 𝑖;

$\bar{w}_{t}$: average wage (contribution base);

$k_{t}$: wage growth rate;

$R_{t}$: contribution rate.

Why summations?

(1) Group-level summation( $\sum_{i=1}^{4} N_{t,x}^{i}$) reflects policy differences across groups;

(2) Age-level summation ($\sum_{x=a_{t}}^{b_{t}-1} N_{t,x}^{i}$) aggregates all labor-age contributors;

(3) Individual differences: Although the model uses average wages and a uniform rate due to data limitations, group classification indirectly captures heterogeneity through differing $N_{t,x}^{i}.$

**5. Data level: macro-aggregation vs. individual data**

The model uses macro-aggregated data rather than micro-level individual records due to data access constraints and research focus:

Data source: National statistics (e.g., MOHRSS, NBS annual reports); contribution base = previous year’s average wage; uniform rate = 20% employer + 8% employee.

Rationale: The study aims to project long-term sustainability trends rather than individual behavioral patterns. Aggregated group-level modeling balances tractability and data availability.

**6. Growth rate of the contribution base**

The growth rate is an assumed parameter based on historical wage growth trends and macroeconomic forecasts, not a government-fixed rate. It is set with reference to:

Historical data: national average wage growth (≈60–80% of GDP growth);

Policy and research: e.g., Lu & Cai (2016);

Scenario variation: adjusted upward under high-fertility scenarios due to higher projected GDP growth.

**7. Pension expenditure: average values vs. actual distribution**

The model adopts a group-average approach rather than assuming any specific distribution (e.g., normality), as Chinese pension distribution is highly skewed:

Government/administrative retirees average 7,600 CNY/month, vs. rural residents’ 245 CNY/month;

Regional disparities: Shanghai ≈ 5,000 CNY, Henan ≈ 3,200 CNY.

The “basic pension” is contributory (minimum 15-year contribution requirement), not a universal flat benefit as in Nordic systems.

**8. Definition of “i-class workers”**

The “i” classification is policy-based, not industry-based:

Focused on reform timing, not sector;

The study covers formal urban employees; informal workers (≈32% of labor force) and unpaid family labor are largely excluded due to lack of contribution records.

**9. Cumulative balance model: why no discounting?**

The actuarial model focuses on cash flow sustainability (deficits, solvency timing) rather than the present value of intergenerational transfers. Discounting may be added in future research to assess intergenerational equity.

**10. Policy implications: delayed retirement**

The study supports gradual retirement age increases as the most feasible reform:

Simulations: delaying retirement by 6 months/year yields a CNY 49.44 trillion surplus by 2050 vs. CNY 147.4 trillion deficit in the baseline.

Policy alignment: consistent with the 2025 Progressive Retirement Law (“gradual increase to 63 for men, 58 for women over 15 years”).

We have revised the manuscript to incorporate these clarifications and aligned the conclusions directly with the stated research questions.
